# Genome-wide DNA methylation status of Mongolians exhibits signs of cellular stress response related to their nomadic lifestyle

**DOI:** 10.1186/s40101-022-00305-0

**Published:** 2022-08-19

**Authors:** Yuta Inaba, Sadahiko Iwamoto, Kazuhiro Nakayama

**Affiliations:** 1 Laboratory of Evolutionary Anthropology, Department of Integrated Biosciences, Graduate School of Frontier Sciences, The University of Tokyo, 5-1-5 Kashiwanoha, Kashiwa, Chiba, 277-8562 Japan; 2 Division of Human Genetics, Center for Molecular Medicine, Jichi Medical University, 3311-1 Yakushiji, Shimotsuke, Tochigi, 329-0498 Japan

**Keywords:** DNA methylation, Mongolians, Livestock, Crop farming, PM20D1, Reactive oxygen species, Obesity, Alzheimer’s disease, Cold adaptation, Sorbitol dehydrogenase

## Abstract

**Background:**

Epigenetics is crucial for connecting environmental stresses with physiological responses in humans. Mongolia, where nomadic livestock pastoralism has been the primal livelihood, has a higher prevalence of various chronic diseases than the surrounding East Asian regions, which are more suitable for crop farming. The genes related to dietary stress and pathogenesis of related disorders may have varying epigenetic statuses among the human populations with diverse dietary cultures. Hence, to understand such epigenetic differences, we conducted a comparative analysis of genome-wide DNA methylation of Mongolians and crop-farming East Asians.

**Methods:**

Genome-wide DNA methylation status of peripheral blood cells (PBCs) from 23 Mongolian adults and 24 Thai adults was determined using the Infinium Human Methylation 450K arrays and analyzed in combination with previously published 450K data of 20 Japanese and 8 Chinese adults. CpG sites/regions differentially methylated between Mongolians and crop-farming East Asians were detected using a linear model adjusted for sex, age, ethnicity, and immune cell heterogeneity on RnBeads software.

**Results:**

Of the quality-controlled 389,454 autosomal CpG sites, 223 CpG sites were significantly differentially methylated among Mongolians and the four crop farming East Asian populations (false discovery rate < 0.05). Analyses focused on gene promoter regions revealed that *PM20D1* (peptidase M20 domain containing 1), which is involved in mitochondrial uncoupling and various processes, including cellular protection from reactive oxygen species (ROS) and thermogenesis, was the top differentially methylated gene. Moreover, gene ontology enrichment analysis revealed that biological processes related to ROS metabolism were overrepresented among the top 1% differentially methylated genes. The promoter regions of these genes were generally hypermethylated in Mongolians, suggesting that the metabolic pathway detoxifying ROS might be globally suppressed in Mongolians, resulting in the high susceptibility of this population to various chronic diseases.

**Conclusions:**

This study showed a significantly diverse DNA methylation status among Mongolians and crop-farming East Asians. Further, we found an association between the differentially methylated genes and various metabolic and neurodegenerative diseases. Knowledge of the epigenetic regulators might help in proper understanding, treatment, and control of such disorders, and physiological adaptation in the future.

**Supplementary Information:**

The online version contains supplementary material available at 10.1186/s40101-022-00305-0.

## Background

Epigenetics refers to the modifications of DNA, DNA-associated proteins, and ribonucleoproteins that do not involve changes in DNA sequences. In mammals, a well-known epigenetic modification, DNA methylation, occurs mainly at a cytosine followed by a guanine (CpG site). Methylation at these CpG sites in or near gene regulatory regions can silence the expression of the relevant genes. Alterations in DNA methylation pattern can induce cell-specific gene expression during development and are thus critical for the diversification of human phenotypes. Cancer, metabolic disorders, and Alzheimer’s disease are closely associated with aberrant DNA methylation. A comparison of genome-wide DNA methylation patterns between rectal cancer and normal rectal epithelial cells showed that the *MGMT* (O6-alkylguanine DNA alkyltransferase) gene promoter has more methylated sites in the cancer cells than in the epithelial cells, resulting in decreased *MGMT* expression and impaired DNA damage repair in the cancer cells [1]. A comparison of DNA methylation patterns on leukocyte-derived DNA between patients with metabolic disorders and healthy individuals showed a significant increase in the DNA methylation level of *ABCG1* (ATP-binding cassette sub-family G member 1), involved in intracellular and extracellular signaling and lipid transport, in the patient’s DNA compared with that in the DNA of healthy individuals [2]. Furthermore, the *PM20D1* (Peptidase M20 domain containing 1) promoter is highly methylated in the postmortem prefrontal neurons of Alzheimer’s disease patients [3].

The DNA methylation level at a particular CpG site is attributed to both genetic and environmental factors. A portion of CpG sites in the human genome showed variable methylation levels that strongly correlated with genotypes of nearby single nucleotide polymorphisms (SNPs) [4–9]. For instance, an obesity-related SNP near *ADCY3* (Adenylate cyclase 3) was correlated with the methylation level of a nearby CpG site that was mapped to an enhancer region of this gene [6]. Further, food-derived nutrients can affect DNA methylation. For instance, low intake of folate, a key source of the one-carbon group in the DNA methylation pathway, is associated with a decrease in genome-wide DNA methylation levels and consequently an increased risk of several diseases [10]. Moreover, epidemiological studies and animal experiments have illustrated that exposure to low or unbalanced nutrition during fetal or early postnatal life increases the risk of lifestyle-related diseases, and DNA methylation is thought to be an underlying mechanism for this phenomenon. A genome-wide DNA methylation analysis of blood cells in individuals who were periconceptionally exposed to famine during the Dutch Hunger Winter revealed variable methylation levels of the genes related to growth and development, such as *INSR* and *CPT1A* for insulin secretion and lipid metabolism, respectively [11]. In addition, in a Greek prepubertal population, a considerable portion of CpG sites had substantially altered methylation levels due to a high lipid diet [12]. Non-nutritional environmental factors, such as temperature and humidity, also affect DNA methylation. In a study of European descents living in Boston, a 5°C increase in the average temperature increased the methylation level of the *ICAM-1* (intercellular adhesion molecule 1) promoter by 9%, and a 10% increase in humidity decreased the methylation level of the same region by 5% [13].

There is a growing body of evidence on the intimate relationship between DNA methylation and human diseases. However, DNA methylation profiles among diverse human populations under long-time exposure to different environmental conditions, and their associations with physiological adaptation, remain to be documented. Heyn et al. evaluated the genome-wide DNA methylation profiles of lymphoblastoid cell lines derived from European, African, and Chinese descendants in the USA and found that a considerable part of the variability of DNA methylation level was due to the SNP genotypes that were highly differentiated among these populations [14]. Further studies, including more diverged or admixed populations, also highlighted the impact of genetic ancestry on the variability of DNA methylation levels rather than environmental factors [15–17]. To provide new insights into the relationship diversity of DNA methylation profiles worldwide, East Asians are worth studying because of their relatively close genetic affinities and their diversified culture, living environments, and phenotypes. These populations have highly different environments and livelihoods, such as nomadic pastoralism in arid and cool regions in the case of Mongolians and agriculture in warm and humid regions in the case of Japanese, Thai, and Chinese populations. In our previous studies, where we compared the various lifestyle-related diseases in East Asian groups with different traditional livelihood, including the Mongolians, Japanese, and Thai, we found that the percentage of obese people was much higher among Mongolians and that the Mongolians maintained lower blood triglyceride levels than the similarly obese individuals in other groups [18–21]. Further, heterogeneity in insulin resistance and visceral adiposity was observed among the various East Asian populations, including Japanese, Korean, and Mongolians [22, 23]. The striking differences in the metabolic traits can be attributed to the dietary differences between Mongolians and other East Asians. Mongolians consume more livestock products and fewer vegetables and fruits. In contrast, Japanese and Thai consume more rice, vegetables, and fish, and fewer meat products [24–27]. Since nutritional status strongly influences DNA methylation levels, we hypothesized that it could lead to a large difference in DNA methylation levels between the Mongolians and the agricultural East Asian populations, such as Japanese and Thai, especially in genes associated with lifestyle-related diseases, such as obesity. Hence, to test this hypothesis, we conducted comparative analyses of genome-wide DNA methylation patterns among the Mongolian, Thai, Japanese, and Chinese populations. Our differential methylation analyses revealed significantly diverse DNA methylation statuses among the Mongolians and crop-farming East Asians. Further, we found an association between the differentially methylated genes and various metabolic and neurodegenerative diseases. Knowledge of the epigenetic regulators might help in proper understanding, treatment, and control of such disorders, and physiological adaptation in the future.

## Methods

### Study participants

This study consisted of 23 Mongolian adult males in Ulaanbaatar and 24 Thai adult males in Bangkok [21]. The mean and standard deviation (SD) of age in each group were 50.4 ± 8.8 years old (y.o.) and 49.5 ±8.4 y.o., respectively. These individuals were healthy and had no apparent medical histories. Peripheral blood cells (PBCs) were collected from these individuals by venous blood sampling for the methylation studies.

### Genome-wide quantification of DNA methylation levels

Genomic DNA was prepared from the buffy coat of the anticoagulated blood samples using the Genetra Puregene kit (Promega, Madison, Wisconsin, USA). Genome-wide quantification of DNA methylation level was performed using the Infinium Human Methylation 450K array (Illumina, San Diego, California, USA). Bisulfite conversion of the DNA samples and acquisition of the intensity data were outsourced to Riken Genesis (Yokohama, Japan). To detect Mongolian-specific changes in DNA methylation status, 450K array data of white blood cells from 8 Chinese adult females (26.3±4.6 y.o.) and 20 Japanese adult males (82.0±8.4 y.o.), whose idat files and information of age, sex, and Sentrix ID information were available in the Gene Expression Omnibus (GSE65638 and GSE151355), were included in the further analyses of the methylome of crop-farming East Asians (CEAs) [28, 29]. For GSE65638, one of each monozygotic twin was randomly selected.

### Processing and analyses of DNA methylation data

Filtering, normalization, white blood cell content estimation, and differential methylation analyses were performed on RnBeads [30]. The details of the filtering procedures are summarized in Supplementary Table 1 of Additional file 1. Against the *β* values of the retained 387,643 sites, background subtraction and normalization were performed using methylumi noob [31] and Dasen [32], respectively. Proportions of immune cells in the specimens were estimated by the LUMP algorithm [33]. Immune cell-type heterogeneity might confound the differential methylation analysis and thus should be adjusted [34]. To this end, contents of eight subpopulations of immune cells, including granulocytes, eosinophils, neutrophils, CD14+ monocytes, CD19+ B cells, CD4+ T cells, CD8+ T cells, and CD56+ natural killer cells in each sample were estimated using a reference methylome data [35]. The global similarity of DNA methylation pattern across samples was visually inspected by principal component analysis (PCA) of the retained 387,643 sites. CpG sites differentially methylated between the Mongolians and CEAs were detected using the linear model method implemented in the RnBeads (limma package). Age, sex, the inferred contributions of the eight immune cell subpopulations, and the origin of data (newly acquired in this study, GSE65638, or GSE151355) were included as covariates in the linear model. The differential methylation analysis was performed against sets of CpG sites clustered in the putative promoter regions of autosomal genes, which included 1.5 kilobases (kb) upstream and 0.5 kb downstream of each transcription start site. False discovery rate (FDR)-adjusted *P* value < 0.05 was set to the significant level. In addition, the combined ranking score of each site, based on the difference in mean methylation levels between groups, the quotient in mean methylation, and the *P* values of the linear model, was also used to evaluate different methylation levels between groups. Genotypes of SNPs in Mongolians and Thai were retrieved from genome-wide SNP genotyping array data obtained in our previous study [36].

### Gene ontology analysis

Gene ontology enrichment analyses were performed using the ClueGO plugin of Cytoscape software [37]. Based on the combined ranking score of the methylation status of promoters, the top 1% of 16,471 autosomal protein-coding genes was selected. Four ontology datasets, including GO, KEGG, REACTOME, and WikiPathway, were included in the enrichment analysis. Two-sided hypergeometric test with the Benjamini-Hochberg correction was applied.

## Results

### Analyses of the global DNA methylation patterns

**Fig. 1:**
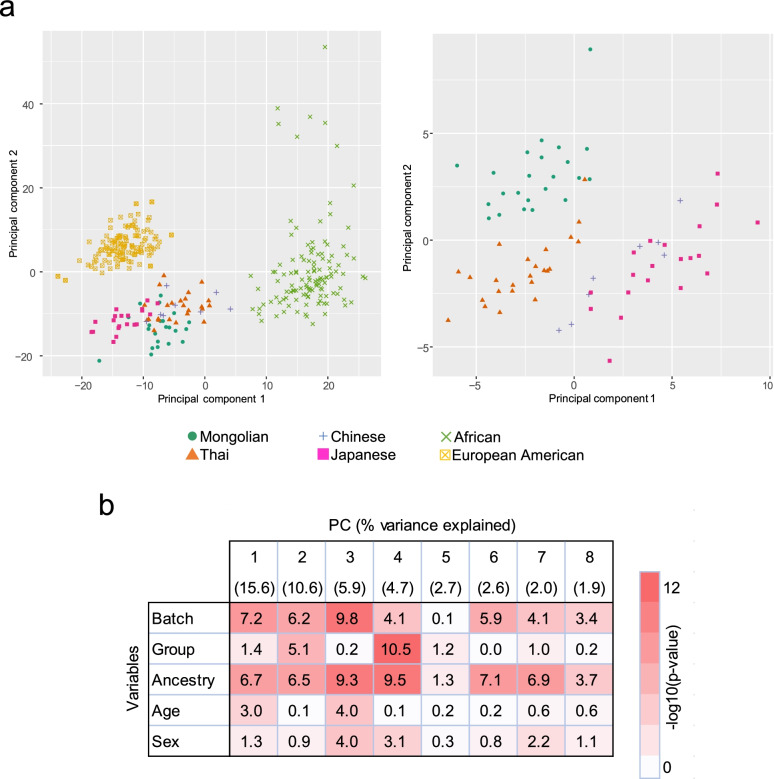
Principal component analysis (PCA) of the global DNA methylation pattern of samples. **a** Left panel shows the result of PCA on global DNA methylation pattern of Asians, Africans, and European Americans [38, 39]. Right panel shows the result of PCA on 7389 highly variable CpG sites in the Asians. Principal components (PC) 1 and 2 are plotted. **b** Association between explanatory variables and the top eight PCs yielded by PCA analysis of 389,454 CpG sites of Asians. For PC1 to PC8, percentages of variance are indicated

We measured the DNA methylation levels of 23 Mongolian and 24 Thai adults using the Infinium Human Methylation 450K arrays and integrated it with the previously published 450K data of blood cells from Chinese and Japanese subjects. After filtering, 389,454 autosomal CpG sites were retained. The mean ± SD values of genome-wide methylation levels and immune cell content estimation of the subjects were 0.556 ± 0.001 and 0.953 ± 0.005, respectively. These results supported the consistency and high proportion of immune cell-derived genomic DNA across the subjects. Next, we performed PCA on the global DNA methylation data of the Asians using 450K datasets of non-Asian PBCs reported elsewhere [38, 39]. Mongolians and Thai formed a single cluster with Japanese and Chinese that were separated from Africans and European Americans (Fig. 1a). The PCA on the 7389 highly variable sites (standard deviation of *β* values across Asian individuals > 0.1) exhibited a tendency for the four ethnic groups to cluster independently (Fig. 1a). We further tested the associations between possible explanatory variables and each eight top principal components (PCs), which explained 46% of the total variance of global methylation levels among the Asians (Fig. 1a). The origin of data (newly acquired in this study, GSE65638, or GSE151355), age, and sex showed a strong association with these PCs (Fig. 1b). We further estimated the contribution rates of immune cell subpopulations in each subject. Of the eight immune cell subpopulations, granulocytes, CD4+ T cells, CD14+ monocytes, and CD19+ B cells showed significant differences in the contribution rates between the populations (Kruskal-Wallis test, *P* < 0.05, see Supplementary Table 2 and Supplementary Figure 1 of Additional file 1). Differential methylation analyses without adjustment for immune cell heterogeneity would yield enrichment of the CpG sites per region that were differentially methylated among the cell subpopulations. Thus, in addition to sex, age, and the origin of data, the contribution rate of the immune cell subpopulations was included as a covariate in the further differential methylation analyses.

### Differences in genome-wide DNA methylation levels at the CpG sites

**Fig. 2:**
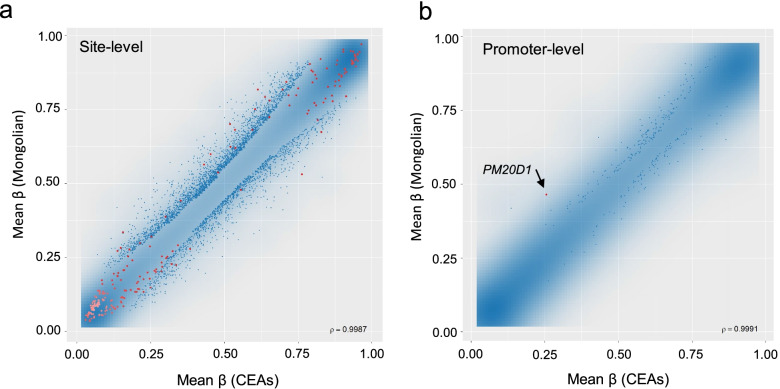
Results of differential methylation analyses. Blue shade indicates the density of CpG sites or regions on each plot. Red dots indicate significant sites or regions after adjustment for false discovery rate (FDR < 0.05) in the linear model. **a** Results of the site-level analysis. **b** Result of the promoter-level analysis. Mean *β* values of CpG sites in the predefined promoter region are shown

Differences in the mean DNA methylation levels of each CpG site among the Mongolians and agricultural East Asian groups are shown in Fig. 2a. A considerable amount of CpG sites were consistently highly methylated or unmethylated across samples. However, such minimal differences would not reflect biologically due to their low *P* value in the linear model. Hence, we concentrated only on the CpG sites with an absolute mean *β* difference > 0.1 (Table 1). Interestingly, several of these ethnically differentiated sites were previously reported for their association with common diseases, for instance, cg09789536 for cow’s milk allergy [40], cg07157834 and cg25629442 for Alzheimer’s disease [41, 42], cg09894276 for obesity [43], and cg04635334 for depression [44]. Moreover, 9 of the 23 CpG sites had a *cis* or *trans* methylation quantitative trait loci [45], suggesting that genome variation plays a non-negligible role in shaping ethnic differences in DNA methylation pattern among closely related populations, as previously validated in inter-continental human populations. To take the magnitude of the difference in methylation level into consideration, we adopted the combined rank approach implemented in RnBeads in further analyses.

**Table 1:** Significant CpG sites (FDR < 0.05)

CpG ID	Mean ***β***			***P*** value	Adjusted ***P*** value	Gene	Region
	Mongolian	CEAs	Difference				
cg09789536	0.278	0.384	−0.106	2.1E−05	4.6E−2	*KLHL17*	Body
cg07157834	0.681	0.535	0.146	1.8E−06	2.4E−2	*PM20D1*	TSS1500
cg07051654	0.781	0.653	0.128	1.9E−05	4.5E−2	*LOC440910*	Body
cg03482534	0.269	0.441	−0.172	2.9E−05	5.0E−2	*NICN1*	TSS200
cg14094333	0.223	0.336	−0.113	2.6E−05	5.0E−2	*KLF*	1st Exon; 5′UTR
cg19526166	0.564	0.431	0.133	1.1E−05	3.8E−2	*TMEM232*	TSS1500
cg05683630	0.600	0.453	0.147	2.3E−05	4.9E−2		
cg17188169	0.844	0.710	0.134	1.0E−05	3.8E−2	*DDX43*	1st Exon; 5′UTR
cg09894276	0.905	0.790	0.115	1.8E−05	4.5E−2	*WDR27*	Body
cg24389034	0.818	0.697	0.121	2.2E−06	2.6E−2	*WDR27*	Body
cg09649198	0.714	0.817	−0.103	2.7E−05	5.0E−2	*PTPRN2*	Body
cg13563298	0.795	0.913	−0.118	9.8E−06	3.8E−2	*WNK2*	Body
cg08344485	0.531	0.763	−0.232	6.0E−07	1.6E−2	*PKNOX2*	5′UTR
cg05909891	0.793	0.637	0.156	2.0E−05	4.6E−2		
cg22805491	0.752	0.604	0.148	3.7E−06	2.9E−2		
cg09208961	0.623	0.521	0.102	2.7E−05	5.0E−2	*LOC91948*	TSS1500
cg04635334	0.282	0.148	0.134	3.8E−08	8.7E−3		
cg27202913	0.272	0.137	0.135	6.2E−07	1.6E−2	*CDH15*	Body
cg08449049	0.335	0.156	0.179	2.4E−06	2.6E−2	*CDH15*	Body
cg25629442	0.147	0.251	−0.104	7.0E−06	3.5E−2	*ENGASE*	TSS200
cg15572907	0.701	0.519	0.182	1.3E−06	2.4E−2	*SPTBN4*	Body
cg15441369	0.818	0.613	0.205	5.2E−06	3.1E−2		
cg26057840	0.674	0.829	−0.155	6.1E−06	3.2E−2		

*FDR* false discovery rate, *CEAs* crop-farming East Asians, *TSS1500* 1500 base pairs (bp) upstream of a transcription start site, *TSS200* 200 bp upstream of a transcription start site, *5′UTR* 5′ untranslated region, *Body* gene region other than that described above

Differential methylation analyses were performed against a series of CpG sites within a promoter region, defined as 1.5 kb upstream and 0.5 kb downstream of a transcription start site (Fig. 2b). Protein-coding genes that were ranked in the top 100 of 28,361 genes with ensemble gene ID are shown in Table 2. *PM20D1*, encoding a secretory enzyme-producing bioactive *N*-acyl amino acids and associated with obesity and neurodegenerative diseases, was the most highly differentiated gene between the Mongolians and CEAs. This gene also cleared the multiple testing correction in the linear model (FDR adjusted *P* < 0.05). The second top gene was *GSTM5* (glutathione S transferase Mu 5), a member of the glutathione S transferase μ family that detoxifies various electrophilic compounds [46]. Additionally, well-known genes involved in energy metabolism, including *SLC16A11* (solute carrier family 16 member 11) [47], *SORD* (sorbitol dehydrogenase) [48], and *G0S2* (G0/G1 switch 2) [49], were also highly differentiated between the Mongolians and CEAs.

**Table 2:** Protein-coding genes in the top 100 combined rank of all autosomal genes

		Mean β^1^						
Gene symbol	# sites	Mongolian	CEAs	Difference^2^	Quotient^3^	# significant site^4^	Lowest *P* values^5^	Rank
*PM20D1*	8	0.465	0.258	0.207	0.904	1	1.8E−06	1
*GSTM5*	5	0.541	0.413	0.129	0.381	0	1.6E−03	6
*RAET1L*	10	0.161	0.105	0.057	0.688	0	2.3E−04	15
*RNF135*	1	0.184	0.129	0.055	0.477	0	1.2E−02	18
*NUDT12*	6	0.157	0.105	0.052	0.543	0	3.8E−04	25
*TRIOBP*	11	0.248	0.199	0.049	0.342	0	3.5E−04	34
*NRIP2*	6	0.511	0.431	0.080	0.266	0	4.6E−03	35
*CHD2*	3	0.307	0.356	−0.050	−0.265	0	7.4E−04	39
*BOLL*	6	0.167	0.103	0.064	0.697	0	8.8E−03	42
*PDE8A*	2	0.187	0.131	0.057	0.531	0	1.5E−03	43
*STMN1*	12	0.429	0.380	0.049	0.270	0	5.1E−03	44
*PRSS22*	11	0.311	0.256	0.055	0.294	0	3.0E−03	45
*TSPAN3*	2	0.177	0.109	0.068	0.662	0	1.4E−02	48
*DPP9*	2	0.164	0.234	−0.070	−0.361	0	3.9E−03	53
*MFSD14B*	1	0.139	0.097	0.041	0.472	0	9.8E−05	55
*SLC17A9*	11	0.301	0.260	0.041	0.306	0	7.6E−04	56
*ITGB1*	5	0.189	0.140	0.049	0.361	0	2.3E−03	60
*SLC16A11*	9	0.189	0.149	0.040	0.365	0	1.1E−03	62
*SORD*	12	0.217	0.177	0.040	0.239	0	1.4E−04	63
*NUDT4B*	2	0.179	0.139	0.040	0.259	0	5.0E−03	66
*IZUMO2*	7	0.349	0.311	0.039	0.212	0	2.1E−03	67
*TSPYL5*	11	0.385	0.347	0.039	0.261	0	2.8E−03	69
*TRMT12*	7	0.210	0.161	0.050	0.562	0	2.8E−02	70
*PABPC3*	3	0.440	0.367	0.073	0.264	0	1.3E−02	72
*G0S2*	12	0.268	0.224	0.044	0.345	0	5.6E−03	76
*SULT1A1*	3	0.395	0.358	0.037	0.529	0	8.7E−03	78
*XPA*	1	0.083	0.119	−0.035	−0.465	0	8.9E−04	82
*RPL22*	4	0.199	0.161	0.038	0.310	0	1.1E−02	83
*ATG4C*	9	0.201	0.149	0.051	0.186	0	1.1E−04	86
*MARVELD2*	8	0.282	0.247	0.034	0.284	0	1.1E−04	88
*RFPL2*	5	0.771	0.682	0.089	0.182	0	1.2E−03	95
*NEIL1*	5	0.482	0.440	0.042	0.181	0	1.6E−02	98
*OOEP*	6	0.881	0.777	0.104	0.180	0	1.0E−05	100

*CEAs* crop-farming East Asians

^1^Regional means of *β* of each site in each group are shown

^2^Regional means of differences in *β* calculated for each site are as follows: mean *β* in Mongolians—mean *β* in CEAs

^3^Regional mean of quotient calculated for each site is as follows: log2 (mean *β* in Mongolians + 0.01/mean *β* in CEAs + 0.01)

^4^Numbers of sites that survived the FDR adjustment are indicated

^5^The lowest *P* values among the sites are indicated

### Hypermethylation of the PM20D1 gene in the Mongolian group

**Fig. 3:**
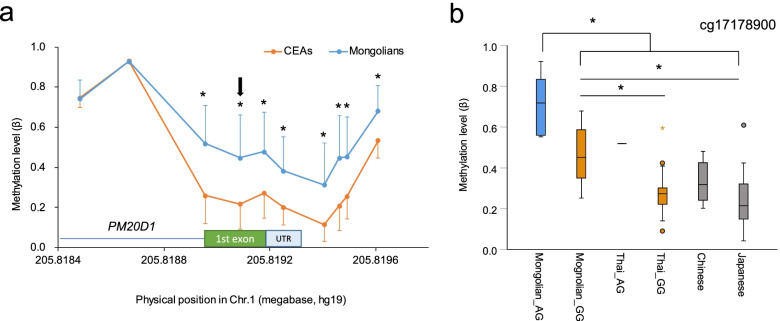
Methylation status of CpG sites in/near the *PM20D1* promoter. **a** Mean and standard deviation values of 10 CpG sites in Mongolians and crop-farming East Asians (CEAs) are shown. The positions of the 5′untranslated region (UTR), 1st exon, and 1st intron of the *PM20D1* are indicated. Position of cg17178900 is shown with an arrow. *P < 0.001 in the linear model. **b** Methylation levels of cg17178900 in different groups are shown. The Mongolian and Thai populations were further grouped according to the genotype of mQTL SNP of cg17178900. Cyan, orange, and gray boxes indicate the methylation level of A/G heterozygotes, G/G homozygotes, and unknown genotype, respectively. *P < 0.05, Tukey’s test

Next, we focused on the methylation status of the putative promoter and gene body of *PM20D1*, the most differentiated gene between the Mongolians and CEAs. Filtration of the 450K data retained 10 CpG sites in this region, and 8 out of the 10 sites were highly methylated in the Mongolians compared with that in CEAs (linear model *P* < 0.05, after Bonferroni correction, Fig. 3a). Methylation levels of each significant CpG site were highly correlated within samples (Spearman’s rank correlation coefficient > 0.85, *P* < 5E−12). Since SNPs 51 kb apart from *PM20D1* acted as methylation quantitative loci (mQTL) for this region [3, 40], we tested rs708727 and cg17178900, one of the SNP-CpG site pair that showed robust associations in Europeans [3, 40]. Genotype frequencies of G/A heterozygous individuals in Mongolians and Thai were 26% and 4%, respectively, and no A/A homozygous individuals were found in these two groups. Consistent with the previous observations in Europeans, G/A heterozygous individuals showed higher methylation levels at cg17178900 than did G/G homozygous individuals. Interestingly, among G/G homozygous individuals, cg17178900 was significantly highly methylated in the Mongolians compared with that in the Thais (Fig. 3b). For other significant CpG sites in this region, G/G homozygote Mongolians also showed higher methylation levels than did G/G homozygote Thais (data not shown). Other known mQTL SNPs of the *PM20D1* promoter were tightly linked with rs708727 in Mongolians as in Europeans but almost absent in Thais, supporting that difference in local linkage disequilibrium (LD) pattern did not explain higher methylation values among G/G homozygous individuals in Mongolians than in Thai.

### Enrichment of metabolic pathways in the top 1% differentially methylated genes

We performed a gene ontology enrichment analysis to determine whether a particular ontology group was overrepresented in a set of genes highly differentiated between the Mongolians and CEAs. Since the combined rank generated by RnBeads included pseudogenes and non-coding RNA genes without enough functional information, we concentrated on the autosomal protein-coding genes. The top 1% genes revealed significant enrichment of biological pathways, for instance, “reactive oxygen species metabolic process,” “nucleotide catabolic process,” “glycolipid biosynthesis process,” “translation repressor activity,” and “negative regulation of Ras protein signal transduction” (see Supplementary Table 3 of Additional file 1). Nearly 80% of the top differentially methylated protein-coding genes were hypermethylated in the Mongolians compared with that in the CEAs. We also applied a method for reference-free cell mixture adjustments to confirm the robustness of the enrichment across different cell type deconvolution models [50]. *PM20D1* and *GSTM5* were the top and the second top loci in the reference-free model, respectively, and moreover, the gene ontology analysis based on the reference-free model supported the overrepresentation of pathways related to nucleotide and reactive oxygen species (ROS) metabolism (FDR-adjusted *P* > 0.0005 and 0.05, respectively).

## Discussion

In this study, we conducted a comprehensive comparison of DNA methylation levels between Mongolians and agricultural East Asians and found that CpG sites in or near the *PM20D1* promoter were differentially methylated. According to previous studies, CpG sites in this region are differentially methylated among human populations worldwide, and distal SNPs acting as mQTL for these sites may result in the methylation differences [14, 51, 52]. The effect of these mQTL SNPs is large, and the SNP rs708727 has an effect size of ~25% in elderly persons [41]. The minor A allele of rs708727, linked to the higher methylation levels of the *PM20D1* promoter, is rare among agricultural East Asians [52] but prevalent in Mongolians. However, in G/G homozygotes of rs708727, methylation levels of the *PM20D1* promoter are significantly higher in Mongolians than in Thais, suggesting that factors other than mQTL SNPs may affect the methylation levels of the *PM20D1* promoter. Further, the *PM20D1* promoter methylation levels showed a modest positive correlation with age in PBCs from Alzheimer’s disease patients [41]. In the present study, Mongolian and Thai samples were adjusted for age, and the Japanese population was more elderly than the Mongolian population but showed lower methylation levels. Thus, factors other than age likely affect the difference in methylation levels observed between the Mongolians and CEAs.

In this study, we hypothesized that the distinctive dietary pattern of Mongolians and CEAs might affect their genome-wide DNA methylation status. Interestingly, the top two differentially methylated genes, *PM20D1* and *GSTM5*, were both involved in cellular responses to ROS [53, 54]. Furthermore, our gene ontology enrichment analyses revealed that the overrepresented ontologies were associated with ROS metabolism. A survey on the blood levels of reactive oxygen metabolites in Asians revealed that middle-aged Mongolians consistently have higher oxidative stress than the age-matched Japanese population [24]. The dietary habit of Mongolians, with high livestock products and fewer fruits, vegetables, and fish, was thought to be a reason for the higher oxidative stress [24–26]. The enrichment of differentially methylated genes related to ROS metabolism indicates that the higher oxidative stress of Mongolians is not only attributed to a low intake of antioxidants (for example, vitamin C) but also alterations in the ROS metabolic pathways induced by imbalanced nutrition. ROS exert multiple adverse effects on human health, and the differential methylation status of the genes involved in ROS pathways might induce a higher disease susceptibility among Mongolians. Moreover, other biological pathways enriched in the highly differentially methylated genes may be related to the different nutritional statuses between the Mongolians and CEAs. For instance, highly ranked differentially methylated genes, namely *NUDT12* and *NUDT4B*, are involved in the nucleotide catabolism pathways and encode members of Nudix hydrolases that catalyze various nucleoside diphosphate analogs, including NAD(P)H, FAD, and coenzyme A, which are essential nucleotide coenzymes [55].

In addition to the global effects on biological pathways, changes in the methylation status of single genes might be related to ethnic differences in susceptibility to polygenic diseases. The methylation status of *PM20D1* is associated with neurodegenerative and metabolic diseases and thus may result in high susceptibility to such diseases among Mongolians. *PM20D1* encodes a multi-functional enzyme producing a series of bioactive *N*-acyl amino acids involved in metabolic regulation [53, 56, 57]. An mQTL SNP in strong LD with rs708727 is associated with body mass index, high-density lipoprotein cholesterol, and insulin resistance in Europeans [46]. The presence of hypermethylated allele and the attenuated expression of *PM20D1* in adipose tissues had a non-favorable effect on these traits [52]. Although it is still unclear how the methylation status of white blood cells and adipose tissues correlate, higher frequencies of the hypermethylated allele and the higher basal methylation level of *PM20D1* in Mongolians correlate with the high prevalence of obesity and related metabolic abnormalities [18–21]. mQTL-dependent *PM20D1* hypermethylation is also associated with Alzheimer’s disease [3]. *PM20D1* methylation level is highly correlated between PBCs and brain tissues [41]. Similarly, as illustrated in a review on the prevalence of Alzheimer’s disease and dietary behaviors, Mongolia has the highest prevalence of Alzheimer’s disease among 10 countries across different continents [58]. Thus, *PM20D1* may mediate adverse effects of environmental risk factors for various diseases through epigenetic modification. Additionally, a recent epigenome-wide association study showed that the *PM20D1* methylation status in PBCs is associated with COVID-19 severity [59]. Further research on the relationship between *PM20D1* methylation levels and various environmental factors may help understand complex gene-environment interactions in the pathogenesis of such diseases.

The cold and arid climate of inland East Asia is likely to have placed strong environmental stress on human physiology. Hence, the inter-group variability in the methylation status may be related to the physiological adaptations to environmental stresses other than nutrition [13]. In mice, knockdown of *Pm20d1* exacerbated glucose homeostasis under high-fat diet challenge but augmented resistance to low temperature independent of known thermogenic programs [56]. Moreover, acute cold exposure altered the expression of *Pm20d1* in peripheral blood mononuclear cells and adipocytes in other mammalian species [60, 61]. The epigenetic changes in *PM20D1* possibly take part in the thermogenic mechanism protecting the human body against cold stress. Another differentially methylated gene, *SORD*, involved in diabetic complications related to the polyol pathway, may participate in cellular adaptation to hypertonicity. Intracellular sorbitol is critical for cell volume regulation in high extracellular osmolality conditions [62], and in Bactrian camels, the renal expression of *SORD* is lower in camels under 24 days of water restriction than in control animals [63]. Similarly, the differential expression of *SORD* may affect renal adaptation to restricted water availability even in humans. Thus, it is interesting to investigate the methylation status of these genes in human tissues more relevant to the physiological adaptation to a cold and arid environment.

## Conclusions

In this study, genome-wide DNA methylation analyses revealed a significantly diverse DNA methylation status even among genetically closely related populations. Further, we found an association between differentially methylated genes and metabolic and neurodegenerative diseases, most likely due to its impact on ROS metabolism pathways. Identification of factors influencing the methylation status of *PM20D1* and other genes identified here would help us understand complex gene-environmental interactions in the pathogenesis of lifestyle-related diseases. There are several limitations to this study. Firstly, the 450K arrays cover limited numbers of CpG sites in the human genome, and thus, data acquisition using methods with higher resolutions, such as the Infinium Methylation EPIC array that interrogates over 850,000 CpG sites or whole-genome bisulfite sequencing, is favorable for a comprehensive understanding of the ethnic differences in DNA methylation pattern. Secondly, sex and age were not matched between the newly acquired and the published data. Although sex and age were adjusted in our linear models, sex and age-matched populations are more suitable to exclude the effects of these confounding factors. Moreover, alternative methods for adjusting immune cell type heterogeneity should be considered. Thirdly, the present differential methylation analysis only focused on the CpG sites near promoter regions, and the impact on more distal CpG sites could not be considered. Lastly, the degree of correlation between the DNA methylation status of PBMCs and other tissues is still unclear. It will be interesting to assess the effect of a dietary component on the DNA methylation status of *PM20D1* and other differentially methylated genes in Mongolians. Moreover, the differentially methylated genes identified here should be tested in other human populations with a long-time history of nomadic lifestyle. Knowledge of the epigenetic regulators might help in proper understanding, treatment, and control of such disorders, and physiological adaptation in the future.

## Supplementary Information

**Additional file 1: Table S1.** Summary of the filtering**. Table S2.** Contribution of immune cell subpopulations estimated from methylome data. **Table S3.** Results of the gene ontology enrichment analysis. **Figure S1.** Principal component analysis (PCA) of global DNA methylation pattern of samples with immune cell references. The plot of component 1 and component 2 is shown. “Reference” indicates the reference methylome data of immune cell subpopulations [35]. **Figure S2.** Methylation status of CpG sites in/near the GSTM5 promoter. Mean and standard deviation of 5 CpG sites in Mongolians and crop-farming East Asians (CEAs) are shown. The positions of the 5′untranslated region (UTR), 1st exon, and 1st intron of the GSTM5 are indicated.

## Data Availability

The present study does not include permission for depositing the genome data into a public database. However, the Infinium 450K datasets used in the current study are available from the corresponding author on reasonable request.

## Abbreviations

ABCG1: ATP binding cassette subfamily G member 1
ADCY3: Adenylate cyclase 3
CEAs: Crop-farming East Asians
CpG: 5′-C-phosphate-G-3′
CPT1A: Carnitine palmitoyltransferase 1
FAD: Flavin adenine dinucleotide
FDR: False discovery rate
G0S2: G0/G1 switch 2
LD: Linkage disequilibrium
GSTM5: Glutathione S-transferase mu 5
ICAM1: Intercellular adhesion molecule 1
INSR: Insulin receptor
MGMT: O-6-Methylguanine-DNA methyltransferase
mQTL: Methylation quantitative trait locus
NADH: Nicotinamide adenine dinucleotide
NUDT12: Nudix hydrolase 12
NUDT4B: Nudix hydrolase 4B
PBC: Peripheral blood cells
PM20D1: Peptidase M20 domain containing 1
ROS: Reactive oxygen species
SD: Standard deviation
SLC16A11: Solute carrier family 16 member 11
SNP: Single-nucleotide polymorphism
SORD: Sorbitol dehydrogenase
