# Supplementary material for: Genome-wide DNA methylation status of Mongolians exhibits signs of cellular stress response related to their nomadic lifestyle
Source: J Physiol Anthropol. 2022 Aug 19;41:30. doi: 10.1186/s40101-022-00305-0 (PMC9388360; doi:10.1186/s40101-022-00305-0)
Supplement: Supplementary file 1 — Additional file 1: Table S1. Summary of the filtering. Table S2. Contribution of immune cell subpopulations estimated from methylome data. Table S3. Results of the gene ontology enrichment analysis. Figure S1. Principal component analysis (PCA) of global DNA methylation pattern of samples with immune cell references. The plot of component 1 and component 2 is shown. “Reference” indicates the reference methylome data of immune cell subpopulations [35]. Figure S2. Methylation status of CpG sites in/near the GSTM5 promoter. Mean and standard deviation of 5 CpG sites in Mongolians and crop-farming East Asians (CEAs) are shown. The positions of the 5′untranslated region (UTR), 1st exon, and 1st intron of the GSTM5 are indicated. [file 40101_2022_305_MOESM1_ESM.pdf]

Supplementary Table 1 Summary of the filtering

Procedures	Numbers of removed sites	Note
Removal of SNP-enriched probes	10,131	filtering out a probe when the last 3 bases in its target sequence overlap with a known SNP
Removal of cross-reactive probes	29,922	RnBeads Default setting
Removal of probe with unreliable measurements (Greedycut)	2,636	RnBeads default setting
Context-specific probe removal	1,384	RnBeads default setting
Removal of probes on sex chromosome	10,189	RnBeads default setting
Removal of probes with many missing values	28	Probes with missing rate ≧ 5% were removed.
Removal of consistent probes	42,106	Probes with beta value standard deviation lower than 0.005 were removed.

Supplementary Table 2 Contribution of immune cell subpopulations estimated from methylome data

Gourp		granulocytes*	CD4Tcells*	CD8Tcells	CD14monocytes*	CD19Bcells*	CD56NKcells	neutrophils	eosinophils
Mongolian	Mean	5.40E-01	1.02E-01	6.86E-19	7.67E-02	5.09E-02	2.16E-01	2.59E-02	4.83E-03
	Standard deviation	1.21E-01	5.55E-02	8.03E-18	3.01E-02	3.11E-02	7.85E-02	8.64E-02	1.92E-02
Thai	Mean	4.43E-01	1.14E-01	3.65E-19	7.77E-02	9.02E-02	2.53E-01	5.40E-02	1.08E-02
	Standard deviation	1.25E-01	5.46E-02	8.69E-18	1.89E-02	2.58E-02	6.86E-02	1.13E-01	2.83E-02
Chinese	Mean	4.51E-01	1.48E-01	-1.04E-18	5.00E-02	9.01E-02	1.97E-01	1.01E-01	1.43E-03
	Standard deviation	1.18E-01	6.05E-02	7.83E-18	1.28E-02	2.35E-02	6.93E-02	1.44E-01	4.05E-03
Japanese	Mean	6.29E-01	3.89E-02	2.37E-03	7.28E-02	3.66E-02	1.94E-01	5.28E-02	8.08E-04
	Standard deviation	1.19E-01	3.87E-02	1.06E-02	3.18E-02	1.78E-02	9.63E-02	1.22E-01	3.61E-03

Asterisks indicate immune cell subpopulations showed significant difference among groups (Krusukal-Wallis test, P < 0.05)

Supplementary Table 3 Results of the gene ontology enrichment analysis

ID	Term	Term PValue Co	GOGroups	% Associated	Nr. Genes	Associated Genes Found
GO:0042552	myelination	4.3E-03	Group00	4.62	6.00	[ADGRG6, AFG3L2, B4GALT6, KLK8, MARVELD1, RARA]
R-HSA:140877	Formation of Fibrin Clot (Clotting Cascade)	7.2E-03	Group01	8.82	3.00	[F2R, GP1BB, PF4V1]
WP:4673	Male infertility	2.6E-02	Group02	3.33	4.00	[CAT, CHD2, PSAT1, REC8]
GO:0060294	cilium movement involved in cell motility	2.2E-02	Group03	3.64	4.00	[CFAP44, PLTP, RSPH9, SORD]
GO:0007286	spermatid development	2.0E-02	Group04	3.18	5.00	[BSPH1, CFAP44, REC8, SBF1, SPINK2]
KEGG:04145	Phagosome	3.1E-02	Group05	3.08	4.00	[ATP6V0A2, DYNC1LI1, ITGB1, MRC2]
KEGG:05140	Leishmaniasis	2.2E-02	Group06	4.55	3.00	[CR1L, ITGB1, MARCKSL1]
KEGG:05204	Chemical carcinogenesis	1.7E-02	Group07	5.45	3.00	[CYP3A5, GSTM5, SULT1A1]
GO:0030371	translation repressor activity	4.4E-03	Group08	12.50	3.00	[CIRBP, EIF4EBP3, RARA]
GO:0045182	translation regulator activity	4.6E-03	Group08	4.76	6.00	[BOLL, CIRBP, EIF4EBP3, RARA, RPL22, RPS9]
GO:0046580	negative regulation of Ras protein signal transduction	5.0E-03	Group09	8.00	4.00	[ITGB1, KCTD13, LZTR1, STMN1]
GO:0035023	regulation of Rho protein signal transduction	1.3E-02	Group09	4.76	4.00	[F2R, ITGB1, KCTD13, STMN1]
GO:0046578	regulation of Ras protein signal transduction	1.7E-02	Group09	3.06	6.00	[F2R, ITGB1, KCTD13, LZTR1, RASGEF1A, STMN1]
GO:0043149	stress fiber assembly	1.8E-02	Group09	3.96	4.00	[ITGB1, KCTD13, PXDN, STMN1]
GO:0072593	reactive oxygen species metabolic process	1.1E-02	Group10	3.18	7.00	[CAT, INAVA, MPV17L, NOX5, PXDN, SLC25A33, SORD]
GO:1903409	reactive oxygen species biosynthetic process	1.5E-02	Group10	5.88	3.00	[INAVA, MPV17L, SLC25A33]
GO:0042743	hydrogen peroxide metabolic process	1.8E-02	Group10	5.26	3.00	[CAT, MPV17L, PXDN]
KEGG:04146	Peroxisome	3.0E-02	Group10	3.80	3.00	[CAT, MPV17L, NUDT12]
GO:0016209	antioxidant activity	4.1E-02	Group10	3.33	3.00	[CAT, PRXL2A, PXDN]
GO:0009166	nucleotide catabolic process	1.0E-03	Group11	9.09	6.00	[NEIL1, NT5C1A, NUDT12, NUDT4B, PDE8A, SORD]
GO:0006195	purine nucleotide catabolic process	4.2E-03	Group11	9.30	4.00	[NT5C1A, NUDT4B, PDE8A, SORD]
GO:0009394	2'-deoxyribonucleotide metabolic process	6.6E-03	Group11	9.38	3.00	[CMPK2, NEIL1, SORD]
GO:0009123	nucleoside monophosphate metabolic process	8.6E-03	Group11	5.63	4.00	[AK1, CMPK2, NT5C1A, SORD]
GO:1901136	carbohydrate derivative catabolic process	1.2E-02	Group11	3.37	6.00	[ARSB, NEIL1, NT5C1A, NUDT4B, PDE8A, SORD]
KEGG:00230	Purine metabolism	2.6E-02	Group11	3.36	4.00	[AK1, NT5C1A, PDE1B, PDE8A]
GO:0016254	preassembly of GPI anchor in ER membrane	3.6E-03	Group12	17.65	3.00	[DPM2, PIGZ, ZNF177]
GO:0006661	phosphatidylinositol biosynthetic process	4.3E-03	Group12	4.09	7.00	[CLSTN1, DPM2, MTMR14, PIGZ, PLEKHA2, SBF1, ZNF177]
GO:0009247	glycolipid biosynthetic process	4.8E-03	Group12	7.69	5.00	[B4GALT6, DPM2, GAL3ST3, PIGZ, ZNF177]
GO:0045017	glycerolipid biosynthetic process	5.2E-03	Group12	3.28	10.00	[ABHD8, CLSTN1, DGKH, DPM2, MTMR14, PIGZ, PLEKHA2, SBF1, SIK1, ZNF177]
R-HSA:163125	Post-translational modification: synthesis of GPI-anchored proteins	1.3E-02	Group12	4.82	4.00	[DPM2, PIGZ, PRSS21, RAET1L]
R-HSA:148325	PI Metabolism	2.5E-02	Group12	4.17	3.00	[MTMR14, PLEKHA2, SBF1]

Supplementary Figure 1

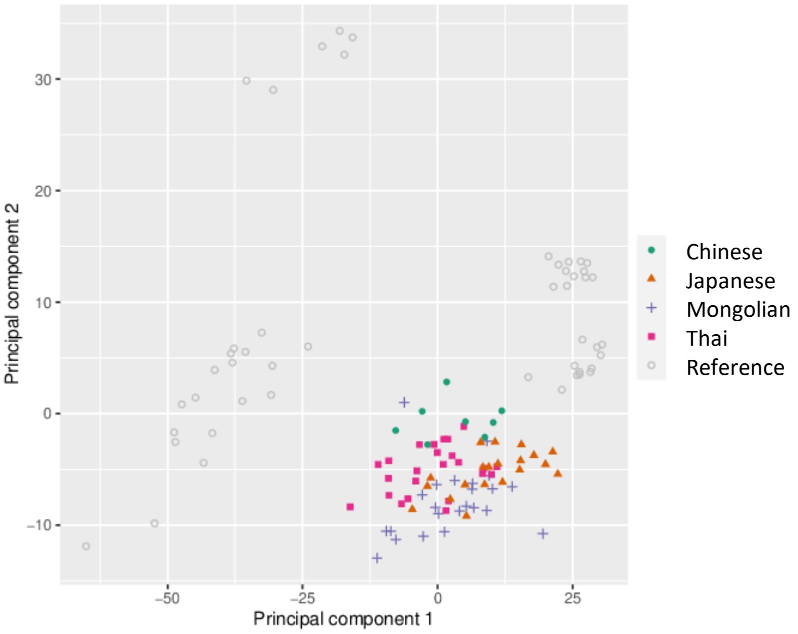

Supplementary Figure 1 Principal component analysis (PCA) of global DNA methylation pattern of samples with immune cell references. The plot of component 1 and component 2 is shown. “Reference” indicates the reference methylome data of immune cell subpopulations [35]

Supplementary Figure 2

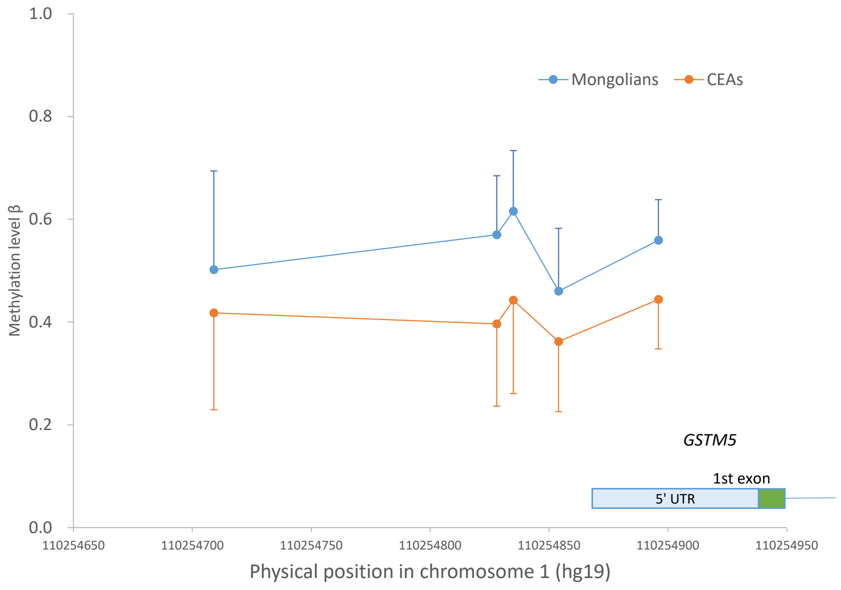

Supplementary Figure 2 **Methylation status of CpG sites in/near the *GSTM5* promoter.**
Mean and standard deviation of 5 CpG sites in Mongolians and crop-farming East Asians (CEAs) are shown. The positions of the 5'untranslated region (UTR), 1st exon, and 1st intron of the *GSTM5* are indicated.
